# Communication with relatives in the ICU

**DOI:** 10.1186/cc9948

**Published:** 2011-03-11

**Authors:** M Liu, S Hutchinson

**Affiliations:** 1 Norfolk & Norwich University Hospital, Norwich, UK

## Introduction

Relatives of patients in the ICU undergo considerable stress. Effective communication with relatives has been shown to: provide support, reduce stress, and improve their well-being and decision-making. Satisfaction also depends on communication by a senior caregiver. Our aim was to determine how well relatives of patients in the Norfolk & Norwich University Hospital (NNUH) ICU are kept informed.

## Methods

The Metavision^® ^Clinical Information System is used for documentation at the 20-bed NNUH ICU. Retrospective data analysis was conducted for patients staying >4 days during 1 October 2009 to 1 January 2010. Data from the 'Relatives Communication' page included: how often and when relatives were first spoken to, and the staff involved. These variables were compared with patient outcome and length of stay on the ICU. During 1 August to 1 October 2010 relatives were asked to anonymously complete a survey evaluating consultations in the ICU.

## Results

Of 64 notes, communication with relatives was documented in 55% of patients. Of these, 60% of communication was conducted by a consultant. More discussions occurred with relatives of patients who died. Increasing duration of stay on the ICU resulted in a higher percentage of relatives being spoken to. Sixty-seven per cent of relatives of patients staying >20 days were not communicated with until after day 4 of admission. Of 40 surveys, all relatives agreed that the patient's condition was discussed with them quickly enough after admission. Ninety-three per cent thought that they were spoken to often enough and 95% felt by the right staff. Eighty per cent were spoken to by senior staff but 45% stated updates were mostly given by nursing staff. Ninety percent felt they were given the right amount of information and in an appropriate location. Ninety percent were satisfied with their consultations. Seventy-three per cent agreed or partially agreed that written information about critical care would have been helpful.

## Conclusions

Analysis of the notes indicated that communication with relatives of patients on the ICU was poor. This prompted surveying relatives' satisfaction directly, which found that most are satisfied with their experiences of communication in the ICU. Hence, we conclude that relatives are well informed - mainly by nursing staff - but documentation of communication requires improvement. The system currently favours recording of formal conversations by medical staff whilst nursing updates are often documented elsewhere. A solution may be to develop a multidisciplinary record of communication.
